# Application of Tamm Plasmon Polaritons and Cavity Modes for Biosensing in the Combined Spectroscopic Ellipsometry and Quartz Crystal Microbalance Method

**DOI:** 10.3390/bios11120501

**Published:** 2021-12-07

**Authors:** Ieva Plikusienė, Ernesta Bužavaitė-Vertelienė, Vincentas Mačiulis, Audrius Valavičius, Almira Ramanavičienė, Zigmas Balevičius

**Affiliations:** 1 State Research Institute Center for Physical Sciences and Technology, Saulėtekio av. 3, 10257 Vilnius, Lithuania; ieva.plikusiene@chgf.vu.lt (I.P.); ernesta.verteliene@ftmc.lt (E.B.-V.); vincentas.maciulis@ftmc.lt (V.M.); audrius.valavicius@ftmc.lt (A.V.); almira.ramanaviciene@chf.vu.lt (A.R.); 2 NanoTechnas—Center of Nanotechnology and Materials Science, Faculty of Chemistry and Geosciences, Vilnius University, Naugarduko Str. 24, 03225 Vilnius, Lithuania

**Keywords:** Tamm plasmon polaritons, cavity mode, spectroscopic ellipsometry, quartz microbalance, biosensors

## Abstract

Low-cost 1D plasmonic photonic structures supporting Tamm plasmon polaritons and cavity modes were employed for optical signal enhancement, modifying the commercially available quartz crystal microbalance with dissipation (QCM-D) sensor chip in a combinatorial spectroscopic ellipsometry and quartz microbalance method. The Tamm plasmon optical state and cavity mode (CM) for the modified mQCM-D sample obtained sensitivity of ellipsometric parameters to RIU of Ψ_TPP_ = 126.78 RIU^−1^ and Δ_TPP_ = 325 RIU^−1^, and Ψ_CM_ = 264 RIU^−1^ and Δ_CM_ = 645 RIU^−1^, respectively. This study shows that Tamm plasmon and cavity modes exhibit about 23 and 49 times better performance of ellipsometric parameters, respectively, for refractive index sensing than standard spectroscopic ellipsometry on a QCM-D sensor chip. It should be noted that for the optical biosensing signal readout, the sensitivity of Tamm plasmon polaritons and cavity modes are comparable with and higher than the standard QCM-D sensor chip. The different origin of Tamm plasmon polaritons (TPP) and cavity mode (CM) provides further advances and can determine whether the surface (TPP) or bulk process (CM) is dominating. The dispersion relation feature of TPP, namely the direct excitation without an additional coupler, allows the possibility to enhance the optical signal on the sensing surface. To the best of our knowledge, this is the first study and application of the TPP and CM in the combinatorial SE-QCM-D method for the enhanced readout of ellipsometric parameters.

## 1. Introduction

The combination of different sensing methods on one platform has become a growing interest for advanced sensing technologies [1,2,3]. The ability to perform simultaneous signal monitoring with different sensing methods on the same platform opens the possibility to gain more precise and extensive information about the investigated process [1,4]. One of the sensing methodologies that combines the two methods on the same sensing platform is spectroscopic ellipsometry (SE) and quartz microbalance with dissipation (QCM-D) method [5,6]. The combination of these two methods has been widely used for studies of thin polymer layers [7,8] and other biological structures. The main advantage of this combined method is that the thickness of the formed layer on the surface of gold can be evaluated by both methods [6].

However, the nanometric gold layers are widely employed for nanophotonic devices with planar profiles [9,10] and plasmonic based optical biosensing that significantly improves the sensitivity of the method [11]. In surface plasmon polariton (SPP)-type optical sensors, a glass prism as a coupler is often used to achieve the conditions of total internal reflection (TIR) that are needed to excite the propagated SPP waves, which are transversal magnetic (TM)-polarized. The exploitation of this SPP phenomenon requires a semitransparent metal film (commonly gold or silver). The more advanced application of surface plasmon resonance is spectroscopic ellipsometry under total internal reflection (TIR) configuration [12,13]. In the total internal reflection ellipsometry (TIRE) method, the measurement of the phase difference between p- and s- polarizations gives much higher sensitivity in comparison with the commercial SPR optical biosensors, where the intensity of only p-polarized light is monitored [14]. However, all advances that give TIR configurations are not available for spectroscopic ellipsometry in combination with QCM-D because SE is used in the conventional configuration and, as a result, the sensitivity of SE part is significantly smaller than plasmon-based optical methods [15,16].

During the last decade, much attention has been given to structures with a thin metal layer covering the top of a 1D photonic crystal (PC). Another type of surface mode, the so-called Tamm plasmon polariton (TPP), appears at the boundary between the photonic crystal and the metal layer [17,18]. TPPs are optical states, which are similar to the electron states proposed by I. Tamm [19], and can occur in the forbidden energy band gap of a 1D photonic crystal. These forbidden energy regions are the stop band of the PCs due to the Bragg reflections in the periodic structure. In contrast to the SPP propagated surface electromagnetic waves (SEWs), the TPPs are non-propagating states and can be excited in both TM and TE polarizations. In fact, the TPP is a standing wave, which is an interference phenomenon of two surface waves propagating in opposite directions [17]. TPPs have an in-plane wave vector, which is less than the wave vector of light in a vacuum, which allows for their direct optical excitation, while for the SPPs to achieve a total internal reflection condition, the incident light has to reach an in-plane wave vector equal to the surface plasmon resonance [20]. Hybrid Tamm plasmon–surface plasmon polariton modes in a strong coupling regime [21] have been also applied for gas [22] and biosensing applications [15]. In this case, the Tamm plasmon component was used for enhanced performance of the SPP component in the TIR configuration. The advantages of the simple [20] excitation configuration of TPP, however, make them useful for enhancing the optical signal at the surface of the modified QCM-D sensor chip for the optical response of ellipsometric parameters.

In this study, the 1D plasmonic nanophotonic structures supporting Tamm plasmon polariton together with cavity modes were generated on the modified QCM-D chip, which was applied for the sensing of protein layer formation. The increased sensitivity of the ellipsometric parameters Ψ (λ) and Δ (λ) due to the excitation of Tamm plasmon polaritons and cavity modes were monitored, analyzed, and compared with that of a conventional SE combined with QCM-D.

## 2. Materials and Methods

### 2.1. Materials

QCM-D sensor discs (QSX 301 Gold) were purchased from Biolin Scientific ABn (Sweden, Gothenburg). Liquids used for the experiments were deionized water, ethanol (99%, Carl Roth GmbH), and PBS pH 7.4. N-Hydroxysuccinimide (NHS) and N-(3-dimethylaminopropyl)-N′-ethyl-carbodiimide hydrochloride (EDC) were purchased from Alfa Aesar (Germany, Karlsruhe). Bovine serum albumin >98% (BSA) was from Carl Roth GmbH. Affinity purified rabbit anti-BSA antibodies were purchased from Immunology Consultants Laboratory, Inc. (Portland, OR, USA). 11-Mercaptoundecanoic acid (11-MUA) and all other chemicals were purchased from Sigma-Aldrich Chemie GmbH (Regensburg, Germany). All salts and other basic chemicals were purchased from Sigma-Aldrich and were of analytical grade.

### 2.2. QCM-D Sensors Discs Modification by Planar Plasmonic Nanophotonic Structure

In this study, two samples were used: the commercially available QCM-D sensor disc (QSX 301 Gold) and an altered mQCM-D sensor disc modified with additionally deposited plasmonic nanophotonic structure (PC/Au). The QCM-D sensor chip consisted of a 300 μm crystal quartz substrate covered by 200 nm Au layer. The PC formed on the top of QCM-D sensor disc was made of alternating TiO_2_ and SiO_2_ layers, produced using the ion beam sputtering (IBS) technique. The one-dimensional photonic crystal (1D PC) consisted of 10 bilayers of TiO_2_/SiO_2_ (60 nm/110 nm). Further, a 40 nm gold layer was sputtered on the top of the PC using the magnetron sputtering (MS) technique.

### 2.3. SE-QCM-D Measurements in the Liquid Ambient 

The measurements were conducted by employing two methods: SE and QCM-D, simultaneously. Ellipsometry measures the amplitude (Ψ) and phase (Δ) parameters for light reflected from a sample, which are related to the reflected field amplitude E_p_ (p-polarization) and E_s_ (s-polarization) of the incident light, by the expression E_p_/E_s_ = tan(Ψ)exp(iΔ). The ellipsometric experiments were conducted using the rotating compensator ellipsometer M-2000X J. A. Woollam (Lincoln, NE, USA) operating in the spectral range from 245 nm to 1000 nm. It should be noted that the SE-QCM-D chamber operates only at one fixed AOI which was equal to θ = 65°. QCM-D measurements were conducted using Biolin Scientific QSense (QCM-D) device QSense Explorer. The QCM-D and mQCM-D sensor chips were placed into a QSense ellipsometry module (QELM 401). Then ellipsometric spectra and resonant frequency of QCM-D sensors discs in air were obtained. After, the cell was filled by deionized water using peristaltic pump (Ismatec IPC4); the cell volume was 100 µL. The flow speed of deionized water was 1.5 mL/min. After 1 min, when the chamber SE/QCM-D was filled by water, the flow was stopped. Subsequently, the cell was filled with ethanol and measurements of ellipsometric parameters were conducted for QCM-D and mQCM-D samples. The ellipsometric measurements of ambient liquids (deionized water and ethanol) were performed to evaluate the sensitivity of QCM-D and mQCM-D to the changes of ambient index of refraction.

### 2.4. QCM-D and mQCM-D Modification with BSA and Anti-BSA

**Figure 1:**
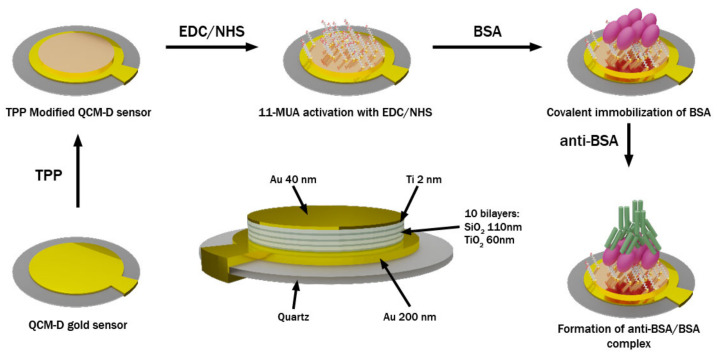
A scheme representing formation of BSA and anti-BSA proteins layers on a modified QCM-D sensor chip.

For the biosensing experiment, both samples (QCM-D and mQCM-D) were cleaned in an ultrasound bath for 3 min, washed with hexane and methanol, and immersed in a solution of 1 mM 11-MUA in methanol for 18 h to allow the formation of a self-assembled monolayer (SAM). The SAM-modified QCM-D and mQCM-D sensor discs were placed in QSense ellipsometry module. The QSense chamber was filled with deionized water and the baseline was established. Then, EDC/NHS solutions mixed in equal parts were injected into the chamber for 15 min to activate the carboxylic groups. During the next step, the chamber was washed with deionized water and filled with PBS pH 7.4. For covalent immobilization of BSA on the 11-MUA SAM, the solution of 1 mg/mL BSA was injected into the chamber. When steady-state conditions were established, the chamber was rinsed with PBS. Finally, 0.5 mg/mL solution of anti-BSA in PBS, pH 7.4 was injected. When the anti-BSA/BSA complex was formed (Figure 1) and steady-state conditions were reached, the chamber was washed with PBS. During all modification steps for both samples (QCM-D and mQCM-D), SE and QCM-D measurements of the kinetics were monitored simultaneously in real time.

## 3. Results and Discussion

### 3.1. Planar Plasmonic Photonic Nanostructure

**Figure 2:**
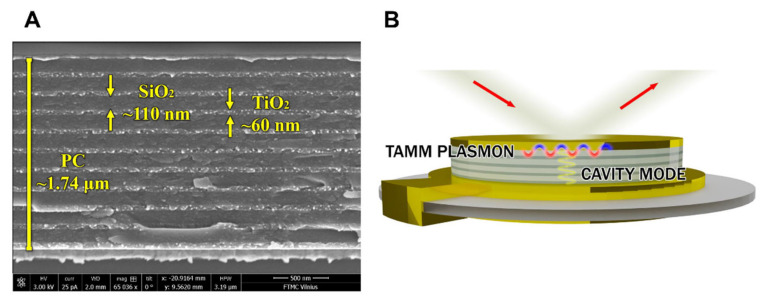
SEM micrograph of the modified QCM-D sensor chip (**A**) and a scheme illustrating formation of BSA and anti-BSA proteins on a modified QCM-D (**B**).

In order to improve the sensitivity of the optical part in the combinatorial SE-QCM-D method, the QCM-D chip was modified by deposition of the Bragg mirror and thin gold layer on the top of thick (200 nm) gold electrode surface. The scanning electronic microscopy (SEM) micrograph and schematic of a modified QCM-D chip are shown in Figure 2A,B, respectively.

**Figure 3:**
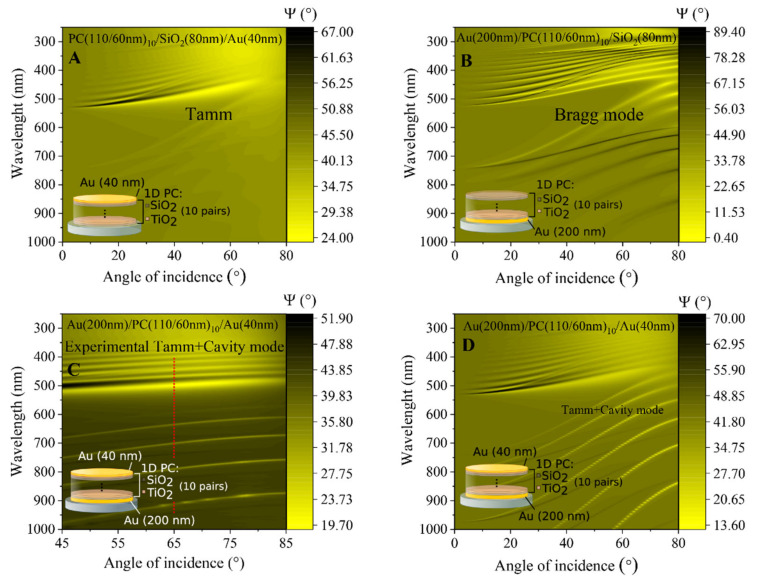
Dispersion relations of calculated TPP (**A**), cavity mode (CM) (**B**), TPP + CM (**D**), and an experimental dispersion of the investigated modified QCM-D sensor chip, supporting TPP and CM (**C**). Red dashed line in (**C**) marks a cross-section at θ_i_ = 65°.

The optical response of ellipsometric parameters Ψ (λ) and Δ (λ) in such a modified structure have shown the presence of the Tamm plasmon polariton optical states and resonant cavity modes in the Bragg mirror between two gold layers. In order to analyze details such as the optical response, the spectroscopic ellipsometry measurements were first conducted in ambient air to determine the optical dispersion of QCM-D sensor chip modified with 1D PC made from ten bilayers of TiO_2_/SiO_2_ and a thin (40 nm) gold layer on the top. Variable angle spectroscopic ellipsometry measurements were performed in a wide range of AOI (45–85°) (Figure 3). In the Ψ ellipsometric parameter map, the dispersion branch starting at 550 nm corresponds to the Tamm plasmon polariton, while the lower periodic branches are attributed to the cavity mode generated in the 1D PC between the two gold mirrors (Figure 3C,D). The rather wide spectral resonance of Tamm plasmon polaritons was observed at 450 nm; meanwhile, the narrow periodic dips of cavity modes manifested themselves at 580 nm, 620 nm, 680 nm, and 800 nm, respectively. To prove the origin of the following optical features in the spectra of ellipsometric parameters, the numerical simulation of the multi-layered structure was conducted. Foremost, the dispersion relation of the 1D PC and thin gold layer were modelled (Figure 3A). The dispersion map of the ellipsometric parameter Ψ (λ, θ) shows the branch of the Tamm plasmon polariton at the 550 nm and very weak oscillations of cavity modes marked with dashed lines. It is clearly seen that such a multi-layered structure generated optical states of Tamm plasmons, which arose at the interface between 1D PC and thin gold layer. In order to analyze the contribution of bottom gold layer to the whole optical response of modified mQCM-D sensor chip, the dispersion relationship of the ellipsometric parameter Ψ (λ, θ) for the substrate/200 nm Au/1D PC was simulated (Figure 3B). As can be seen, noticeable enhancement of Bragg oscillations appears due to the bottom gold layer, while the absence of the thin gold layer on the top eliminates the Tamm plasmon polaritons excitation. The yellow and black branches correspond to the p- and s-polarizations, respectively. Finally, the numerical simulation was conducted with a multi-layered structure (Figure 3D), which was used for experimental measurements with both gold layers (Figure 3C). The map of dispersion relation follows the measured experimental dispersion relation and both the optical effect of the Tamm plasmon polariton and the Bragg modes that were simulated in Figure 3A,B were clearly recognizable in the measured dispersion map of Figure 3C.

In order to compare the optical signal sensitivity of standard QCM-D sensor chip covered by 200 nm gold (QCM-D) and modified QCM-D sensor chip supporting Tamm plasmon polariton and cavity modes, both were tested by varying the refractive index of liquid ambient by changing deionized water to the ethanol in the SE-QCM-D chamber for measurements in liquids. This chamber was filled with high purity deionized water and spectra of ellipsometric parameters Ψ (λ) and Δ (λ) were measured for both samples, standard QCM-D sensor chip and modified QCM-D/TPP + CM at the AOI = 65°. Firstly, the chamber was filled with high purity water which acted as a bulk media having refractive index *n* = 1.333 at λ = 600 nm. After that, high-purity deionized water was changed to ethanol bulk medium, which has a refractive index of *n* = 1.361 at λ = 600 nm.

**Figure 4:**
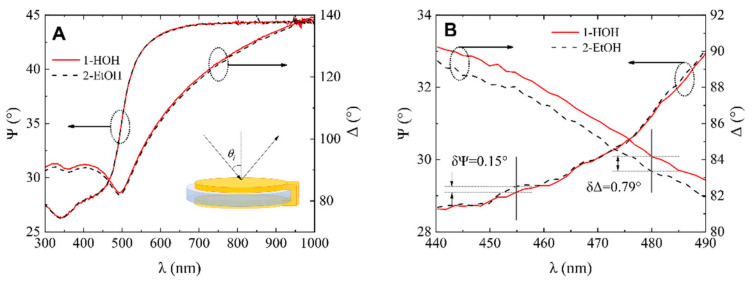
(**A**) Ellipsometric parameter dependence on wavelength λ for (1) QCM-D sensor disc in water and (2) QCM-D chip in ethanol, (**B**) zoomed view of ellipsometric parameters Ψ and Δ dependence on wavelength λ for (1) QCM-D chip in water (red curves) and (2) QCM-D chip in ethanol (dashed curves) with sensitivity parameters δΨ and δΔ.

Spectroscopic ellipsometry measurements have shown that for the standard QCM-D sensor chip, the changes in the ellipsometric parameters Ψ (λ) and Δ (λ) due to variation of refractive index of ambient liquids was miniscule. The differences registered at λ = 455 nm wavelength, was equal for δΨ = 0.15 and δΔ = 0.79. No clear differences for the ellipsometric parameter Ψ values were observed between curves 1 and 2 in Figure 4A. The inset (close-up view) of Figure 4A is presented in Figure 4B. For the ellipsometric parameter Δ, the difference between high-purity water and ethanol (Figure 4A) was registered as 84.15° and 83.36° at λ = 480 nm wavelength for high-purity water and ethanol, respectively (insets Figure 4B).

**Figure 5:**
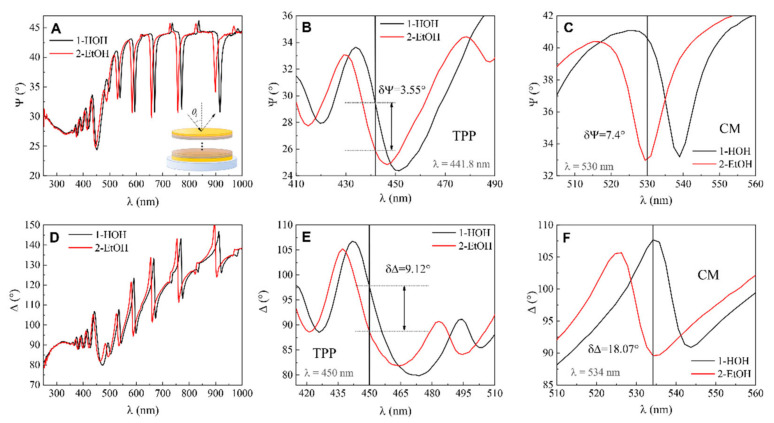
(**A**) Ellipsometric parameter Ψ dependence on wavelength λ for (1) mQCM-D in water-filled cell, (2) QCM-D/TPP in ethanol-filled cell, and zoomed view of A for TPP (**B**) and CM (**C**) components. (**D**) Ellipsometric parameter Δ dependence on wavelength λ for (1) QCM-D/TPP in water-filled cell, (2) QCM-D/TPP in ethanol-filled cell, and zoomed view of D for TPP (**E**) and CM (**F**) components.

Further, the same spectroscopic ellipsometry measurements were conducted for modified QCM-D/TPP + CM sensor chip. The optical response of ellipsometric parameters Ψ (λ) and Δ (λ) showed that the dip in the Tamm plasmon polariton observed at 451 nm for Ψ (λ) in the water and blue-shifted to 446 nm in the ethanol (Figure 5A,B). At 441.8 nm Ψ obtained for water was 29.47° and for ethanol 25.92°, respectively. The calculated difference values at 441.8 nm were δΨ = 25.92° − 25.47° = 3.55°. For ellipsometric parameter Δ, the Tamm plasmon polariton was observed at 465 nm and 475 nm in water and ethanol, respectively (Figure 5D,E). The difference in the ellipsometric parameter δΔ = 98.01° − 88.89° at λ = 450 nm was 9.12°.

As the modified mQCM-D structure also supports cavity modes together with TPP, it makes it possible to employ these modes for sensing refractive index changes. In the spectra of ellipsometric parameters, the cavity modes manifested themselves as the narrow dips in the Ψ (λ) and abrupt changes of Δ (λ) in the vicinity of resonance, respectively (Figure 5C,F).

The difference in ellipsometric parameter Ψ values at λ = 530 nm between water- and ethanol-filled SE-QCM-D chambers was estimated to be δΨ = 7.4°. The value of ellipsometric parameter Δ for cavity mode in high-purity water at λ = 534 nm was 107.67° and in ethanol was 89.6°. The difference was δΔ = 18.07°.

The difference in refractive index between the two bulk media was n_(Eth)_ − n_(H2O)_ = 1.361 − 1.333 = 0.028. After that, sensitivity to refractive index unit (RIU) of ellipsometric parameters for standard QCM-D sample, mQCM-D/TPP, and mQCM-D/CM samples were evaluated. The sensitivity data are presented in Table 1.

In the case of the standard QCM-D sample, the calculated sensitivity was Ψ = 5.35 RIU^−1^, Δ sensitivity was Δ = 28.21 RIU^−1^. For the TPP component in the modified mQCM-D sample, the sensitivity of ellipsometric parameters to RIU was Ψ_TPP_ = 126.78 RIU^−1^ and Δ_TPP_ = 325 RIU^−1^. For cavity mode in modified mQCM-D, the sensitivity Ψ_CM_ = 264 RIU^−1^ and Δ_CM_ = 645 RIU^−1^. The ellipsometric parameter Ψ_TPP_ sensitivity was 27.3 times higher and Ψ_CM_ sensitivity was 49.3 times higher in comparison to the standard Ψ sensitivity to RIU. The Ψ_CM_ was 2.08 times more sensitive to bulk RIU changes than Ψ_TPP_. The relative sensitivity of the ellipsometric parameter Δ was 11.5 higher in the case of Δ_TPP_ and 22.86 times higher in the case of Δ_CM_ than standard Δ. The ellipsometric parameter of cavity mode Δ_CM_ was about two times more sensitive than Δ_TPP_, similarly to the compared sensitivities of the ellipsometric parameter Ψ. In this study, the main focus was on the enhanced sensitivity performance of ellipsometric parameters compared with standard SE-QCM method; however, the spectral shift of the TPP and cavity mode resonances was also registered when the ambient liquid was changed from water to ethanol, and for TPP it was about 10 nm; meanwhile for cavity mode, there was a shift of about 9 nm to a longer wavelength. Such a spectral shift gives sensitivity of 352 nm/RIU for TPP and 321 nm/RIU for cavity mode, respectively. The comparison of refractive index sensing of various nanophotonic structures is presented in the Table 2.

### 3.2. Application for Biosensing

**Figure 6:**
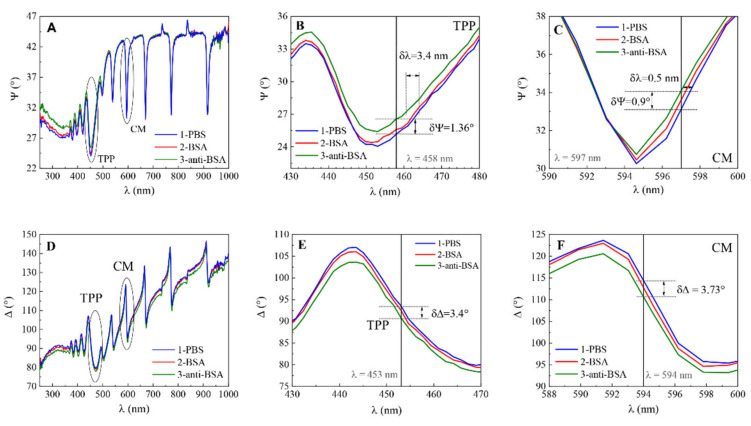
Spectra of ellipsometric parameters Ψ vs. λ for (**A**): 1-PBS, 2-BSA, 3-anti-BSA, (**B**,**C**) zoomed view and Δ vs. λ for (**D**): 1-PBS, 2-BSA, 3-anti-BSA, (**E**,**F**) zoomed view.

Protein adsorption at solid surfaces plays an important role in many natural processes and thus is of huge interest in research various areas including medicine, pharmaceutical sciences, analytical sciences, biotechnology, cell biology, or biophysics [27]. To demonstrate the capability of planar plasmonic nanophotonic structures for biosensing applications, bovine serum albumin (BSA) monolayer was formed on 11-MUA modified surface of QCM-D and mQCM-D. In addition, an affinity interaction with a specific antibody, anti-BSA, was established. In all steps, the ellipsometric parameters Ψ and Δ vs. wavelength were recorded and presented in Figure 6. It was shown before [5] that the combination of electrostatic and hydrophobic interaction, which is supported by the 11-MUA self-assembled monolayer, allows the attachment of more BSA protein on the surface than with other SAMs. It was also reported [26] that a graphene oxide monolayer instead of SAMs on the gold surface can significantly improve the SPR signal response for biosensing. To evaluate the sensitivity and perform detailed analysis, the magnified view of ellipsometric parameter Ψ for TPP and cavity mode for modified sensor chip mQCM-D was determined (Figure 6B,C for TPP and E, F for cavity mode). The optical response of ellipsometric parameter δΨ detected for TPP mode between curves 1 and 3 was 1.36° (Figure 6B), while for CM mode the difference was δΨ = 0.9° (Figure 6C). In addition, the simultaneously registered ellipsometric parameter Δ showed the difference δΔ between curves 1 and 3 for TPP was 3.4° (Figure 6E) and 3.73° for cavity mode (Figure 6F), respectively.

The spectroscopic ellipsometry data for the modified mQCM-D sensor chip were analyzed by a multilayer model [28,29,30]. In this study, the multilayer model represented the following structure: Quartz substr/Au (200 nm)/1D PC (TiO_2_/SiO_2_ (110/60 nm)/Au (40 nm)/MUA-11/BSA+anti-BSA/buffer solution. First, the structure with pure gold layer/buffer interface without proteins, was analyzed to evaluate the reference optical properties of the multilayer structure, such as the thickness and optical constants of each layer. At the starting point of the fitting the optical constants of the materials were used, namely BK7 [31], SiO_2_ [32], Au [33] and TiO_2_ [34] were taken from the literature. Reasonably good fitting results (MSE = 11.3) were obtained; only the thicknesses of the multilayer structure were free fitting parameters, while the optical constants stayed fixed. Furthermore, the ellipsometric spectra of attached BSA and anti-BSA proteins on the gold surface were analyzed, additionally introducing the layer describing the attached surface mass of the studied proteins. The refractive index of the protein layer was approximated as a homogeneous layer and described using the Bruggeman effective medium approximation (EMA). The EMA considers the BSA+anti-BSA proteins layer to be an isotropic physical mixture of two elements, protein and buffer solution, and homogenous on the scale of wavelength. The effective refractive index of the mixture was calculated from the volume fractions of its components, assuming that these retain their intrinsic optical properties and the thickness of the layer was obtained d = 18 nm.

While the thickness and effective refractive index of BSA proteins were determined from regression analysis, de Feijter’s formula [35] $\Gamma =\frac{d\left(n-n_{buffer}\right)}{dn/dC}\times 100$ allows the evaluation of the surface mass (ng/cm^2^), where *dn/dC* = 0.18 $\frac{cm^{3}}{g}$ [36] is the refractive index increment for the layer material, depending on the protein concentration in the buffered solution, d is the thickness (nm), and n is the refractive index of the protein layer obtained from regression analysis and *n_buffer_* is the refractive index of the buffered solution. The attached surface mass for spectroscopic ellipsometry Γ_SE_ ≈ 600 ng/cm^2^ was calculated for the resonant wavelength of Tamm plasmon polariton and cavity mode excitation, meanwhile the obtained surface mass from the QCM-D was Γ_QCM-D_ ≈ 1750 ng/cm^2^. Such a differences in attached surface mass is explained by contribution of buffer solution to the mass evaluation in QCM-D method, meanwhile from the SE evaluated the “dry mass” of the protein [5]. Such information obtained simultaneously from the coupled methods opens the possibility of studying protein conformational changes depending on the pH, surface charge, and many other factors [37,38].

It should be noted that in the case of biomolecule interaction, the sensitivity of the ellipsometric parameters of Tamm plasmon polariton was higher for Ψ and, generally, had the same sensitivity for Δ compared with cavity modes. The ellipsometric parameter of TPP was Ψ = 1.36/0.9 ≈ 1.5 more sensitive to the attached surface mass than cavity mode, meanwhile, the phase difference Δ = 3.4/3.7 ≈ 0.92 was still slightly better for the cavity mode. However, as can be seen from Table 1, the measurements for ambient with different refractive indices showed much higher sensitivity for the cavity mode than for Tamm plasmon polariton. This can be explained by the different origin of Tamm plasmon polaritons and cavity modes. Tamm plasmon polariton in fact is a surface optical state which appears at the interface between the metal film and photonic crystal, meanwhile the cavity modes are generated through the full length of the photonic crystal between the two gold mirrors. Thus, when some changes of refractive index occur on the surface of a thin gold layer (formation of protein monolayer, for instance), this process has more influence for the surface sensitive resonance–Tamm plasmon polariton, than for cavity mode. On the contrary, when the refractive index of the ambient was changed, the angle of light incidence to the gold film surface refracted through the length of the bulk ambient and transmitted to the photonic crystal at a deviate angle, which has a significant influence on the resonant wavelength of the cavity modes. This assumption supports the stronger dependence of the cavity modes on the angle of incidence that can be seen in the dispersion relation map of Ψ ellipsometric parameter (Figure 3).

**Figure 7:**
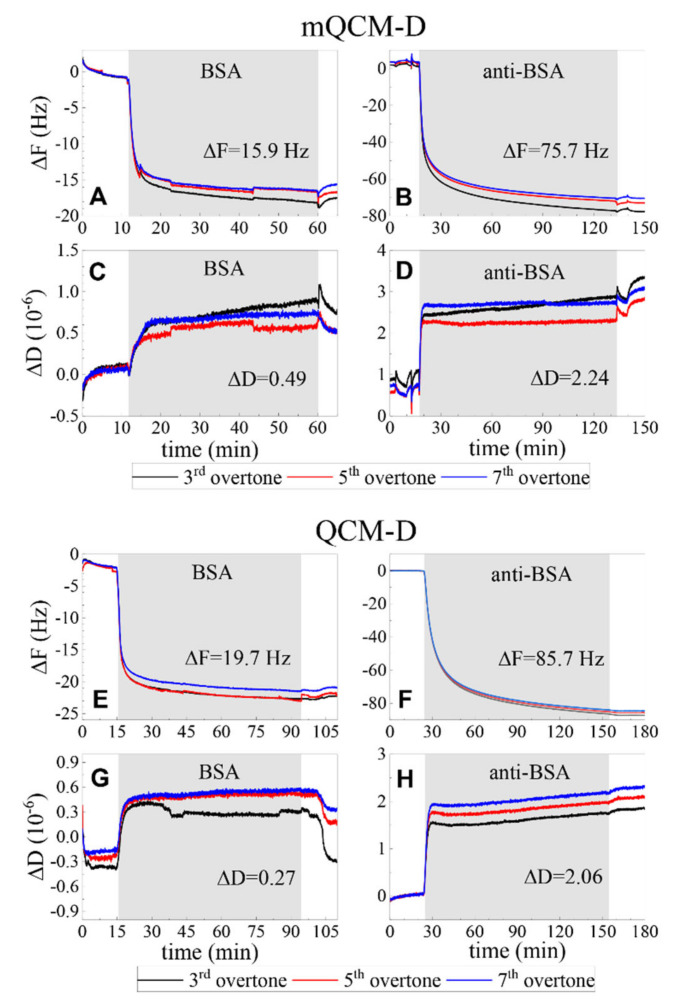
The BSA immobilization and anti-BSA affinity kinetics of modified mQCM-D (**A**–**D**) and QCM-D (**E**–**H**) sensor chips for changes in resonance frequency shift ΔF (**A**,**B**,**E**,**F**) and energy dissipation ΔD (**C**,**D**,**G**,**H**).

In order to clarify the contribution of deposited planar plasmonic photonic structure on the resonant frequency of quartz crystal microbalance the standard and modified QCM-D sensor discs frequencies were measured. The quartz crystal resonant frequency (F1) in deionized water for the standard QCM-D sensor disc was 4.952 MHz. The PC/Au modified mQCM-D sensor chip f1 frequency was equal to 4.922 MHz. The shift of QCM-D frequency in kinetics measurements was consistent between the standard QCM-D and mQCM sensors; thus, the difference of 0.03 MHz in f1 did not affect the sensitivity of QCM-D method. Figure 7 presents the covalent BSA immobilization on the self-assembling monolayer 11-MUA and affinity interaction of anti-BSA with formed BSA monolayer on both samples: mQCM-D (Figure 7A–D) and QCM-D (Figure 7E–H), A–D, and E–H demonstrates the ΔD and ΔF of the binding kinetics between proteins. As it can be seen from Figure 7, the resonance frequency shift ΔF and energy dissipation ΔD were similar for both samples (mQCM-D and QCM-D). The performance of both modified and standard QCM sensor chips was similar.

## 4. Conclusions

The planar plasmonic photonic structures made from periodic dielectric layers and thin metal film were designed for improved performance of ellipsometric parameters Ψ (λ) and Δ (λ) in the combinatorial spectroscopic ellipsometry and quartz microbalance method. The low-cost 1D plasmonic photonic structures supporting Tamm plasmon polaritons and cavity modes were employed for the optical signal enhancement by modifying the commercially available QCM-D sensor chip.

This study has shown that for refractive index sensing of ambient liquids, the planar plasmonic photonic nanostructures exhibit higher sensitivity than conventional quartz microbalance sensor chip, namely the cavity modes and Tamm plasmon polariton were about 49 and 24 times more sensitive than the standard QCM-D sensor chip, respectively. For the ambient refractive index sensing, the cavity modes showed two-fold better sensitivity for ellipsometric parameters Ψ (λ) and Δ (λ) in the Tamm plasmon polariton mode. However, the TPP still showed about 24 and 12 times higher sensitivity for the ellipsometric parameters Ψ and Δ, respectively, than the standard QCM-D sensor chip. Meanwhile, for the optical biosensing signal readout, the sensitivity of Tamm plasmon polaritons and cavity modes was comparable and higher than that of the standard QCM-D sensor chip. This fact can be explained by the different origin of Tamm plasmon polaritons and cavity mode phenomena, the first of which is a surface optical state, and the second one is generated through the full length of the cavity. This difference in optical features gives additional advances and can determine whether the surface or bulk process is dominant. The dispersion relation feature of Tamm plasmon polaritons, namely the direct excitation without additional coupler, makes it possible to enhance the optical signal on the sensing surface, despite the fact that Tamm plasmons are excited at the inner interface of the gold layer. The generation of Tamm plasmon polaritons at the inner interface is not optimal for the sensing applications; however, in this optical configuration, TPP gives about 24 times improved sensitivity in the Ψ ellipsometric parameter. To the best of our knowledge, this is the first study to apply Tamm plasmon polaritons and cavity mode in a combinatorial SE-QCM-D method for the enhanced readout of ellipsometric parameters.

## Figures and Tables

**Table 1:** Sensitivity and relative sensitivity of ellipsometric parameters changes to refractive index unit (RIU).

Sensitivity	
Ψ = 0.15/0.028 = 5.35 RIU^−1^	Δ = 0.79/0.028 = 28.21 RIU^−1^
Ψ_TPP_ = 3.55/0.028 = 126.78 RIU^−1^	Δ_TPP_ = 9.12/0.028 = 325 RIU^−1^
Ψ_CM_ = 7.4/0.028 = 264 RIU^−1^	Δ_CM_ = 18.07/0.028 = 645 RIU^−1^
**Relative Sensitivity**	
Ψ_TPP_/Ψ = 23.7	Δ_TPP_/Δ= 11.5
Ψ_CM_/Ψ_TPP_ = 2.08	Δ_CM_/Δ_TPP_ = 1.98
Ψ_CM_/Ψ = 49.3	Δ_CM_/Δ= 22.86

**Table 2:** Comparison of various nanophotonic structures for refractive index sensing.

Method	Material Used	Sensitivity	Reference
Tamm plasmons	Porous Si 1D PC/Au	139 nm/RIU	[23]
Fabry–Perot cavity	Porous Si	140 nm/RIU	[24]
Photonic crystal nanostructures	Free standing silicon membrane	103 nm/RIU	[25]
Tamm plasmons	Gold coated nanoporous alumina PC	106 nm/RIU	[26]
Hybrid Tamm and surface plasmons in strong coupling	TiO_2_/SiO_2_ 1D PC/Au TIRE 5 bilayers	3200 nm/RIU	[16]
This study			
Tamm plasmons	TiO_2_/SiO_2_ 1D PC/Au 10 bilayers	352 nm/RIU	
Cavity mode	TiO_2_/SiO_2_ 1D PC/Au 10 bilayers	321 nm/RIU	

## Data Availability

Not applicable.
